# THE SHARED MEANING OF COMPASSION FATIGUE AMONG REGISTERED NURSES WORKING IN SKILLED NURSING FACILITIES

**DOI:** 10.1093/geroni/igz038.2586

**Published:** 2019-11-08

**Authors:** Marlene Steinheiser

**Affiliations:** Infusion Nurses Society, Norwood, Massachusetts, United States

## Abstract

The purpose of this hermeneutic interpretive phenomenology study was to describe the shared meaning of compassion fatigue (CF) among registered nurses (RNs) who work in skilled nursing facilities (SNFs). The specific aims were to describe: 1) contributors (triggers, situation, or patient characteristics) that cause symptoms of compassion fatigue, 2) associated physical and emotional symptoms, and 3) the short-term outcomes of unresolved compassion fatigue impacting nurses and patient care. CF can negatively impact patient outcomes, is associated with decreased quality of patient care, and can be a reason why nurses leave the profession. Eight participants were interviewed three times each, while concurrent data analysis helped to formulate mutual understanding of the phenomenon and informed subsequent interviews. Self-reflection, journaling, record keeping, and use of direct quotes enhanced trustworthiness. Four shared meanings were abstracted:1) I feel conflicted and that causes my CF; 2) physical and emotional manifestations of CF; 3) CF is infused in every aspect of my life; 4) we are trying to cope with CF. The participants shared their central desire to make a difference in the lives of their patients, which was of paramount importance. When participants felt they were unable to make the desired difference, they began to develop symptoms of CF. Symptoms were compounded when they experienced frequent patient deaths. A resiliency program specifically addressing the needs of SNF nurses, incorporating individuals and their organizations, could positively impact the nurses’ professional quality of life. Future research is needed to better understand CF and interventions specific to SNF nurses.

